# Antiinflammatory Activity of a Polyherbal Formulation

**DOI:** 10.4103/0250-474X.49123

**Published:** 2008

**Authors:** S. R. Deorukhakar, A. Dethe, R. R. Vohra, R. Ghosh, J. Chaudhary, V. Kadam

**Affiliations:** Department of Pharmacology, Bharati Vidyapeeth's College of Pharmacy, Navi Mumbai-400 614, India; 1 Exotic Naturals, 118 Mourya House, Link Road, Andheri (W), Mumbai-400 053, India

**Keywords:** Entox^®^, carrageenan, cotton pellet granuloma, antiinflammatory, polyherbal, formulation

## Abstract

The antiinflammatory activity of the polyherbal formulation Entox^®^ was investigated in rats for acute and sub acute models of inflammation using carrageenan-induced rat paw edema and cotton pellet granuloma methods respectively at a dose of 300 mg/kg and 600 mg/kg administered orally. The formulation in doses of 300 mg/kg and 600 mg/kg showed 51.61% and 54.84% inhibition of paw edema, respectively at the end of 3 h. The percent inhibition of granuloma by cotton pellet method was 27.92% and 53.17%, respectively. The formulation showed a significant antiinflammatory activity in both the experimental models and the activity was comparable to that of the standard drug, indomethacin.

The formulation, Entox^®^ is constituted of fruits of *Terminalia chebula* (Combretaceae) and *Embelica officinalis* (Euphorbiaceae), fruit rind of *Punica granatum* (Punicaceae), bark of *Terminalia arjuna* (Combretaceae), roots of *Rubia cordifolia* (Rubiaceae), *Withania somnifera* (Solanaceae) and *Tinospora cordifolia* (Menispermaceae) and rhizomes of *Curcuma longa* (Zingiberaceae). Entox^®^ is currently being used as an antioxidant formulation. The studies have shown that oxidation is one of the mechanisms in the inflammatory process^1^. The antiinflammatory activity of each of the constituents of this formulation which includes *Terminalia chebula*^2^, *Emblica officinalis*^3^, *Punica granatum*^4^, *Terminalia arjuna*^5^, *Rubia cordifolia*^6^, *Withania somnifera*^7^, *Tinospora cordifolia*^8^ and *Curcuma longa*^9^ has been reported in scientific literature. Hence the present study was designed to study the antiinflammatory activity of all the constituents when combined together.

The antiinflammatory activity was evaluated in male albino rats of Wistar strain (150-200 g) by both carrageenan-induced rat paw edema and cotton pellet granuloma method. All the experimental protocols were approved by Institutional Animal Ethics Committee (IAEC). The rats were given a standard laboratory diet and water *ad libitum*. In carrageenan-induced rat paw edema method, the animals were divided into 4 groups of 6 animals each. Food was withdrawn 16 h before drug treatment. The first group received 0.5 ml of 1% gum acacia solution orally and served as a control. The second group of animals was administered indomethacin (10 mg/kg, *p.o.*). The animals of groups 3 and 4 were treated with the formulation (300 mg/kg and 600 mg/kg, orally). Acute inflammation was produced by sub plantar injection of 0.1 ml of 1% suspension of carrageenan in normal saline, in the left hind paw of the rats, 1 h after oral drug treatment. The paw volume was measured plethysmometrically at 0, 1, 2, 3 and 4 h after the carrageenan injection. The difference in initial and the subsequent reading gave the actual edema volume and the percent inhibition was calculated^10^,^11^ (Table 1). The data was analyzed using one way ANOVA followed by Dunnett's test and p<0.01 was considered significant. In cotton pellet granuloma method, sterile cotton pellets (30±1 mg) were implanted subcutaneously bilaterally in the groin region under light ether anesthesia. The animals were divided into 4 groups of 6 animals each and were treated consecutively for 7 d. The treatment for all four groups was the same as that mentioned in the earlier method except that indomethacin (5 mg/kg, orally) was administered to second group. The animals were sacrificed on the 8^th^ d. The granulation tissue with cotton pellets was removed and dried overnight at 60° to constant weight. The difference between dry weight of dissected cotton pellet and the weight of individual cotton pellet before implantation gave the dry weight of granuloma^11^,^12^ (Table 2). The data was analyzed using one way ANOVA followed by Dunnett's test and p<0.01 was considered significant.

**TABLE 1:** EFFECT OF FORMULATION ON CARRAGEENAN –INDUCED RAT PAW EDEMA

Time (h)	Volume of edema (ml)			
	
	Control (Vehicle, *p.o.*)	Indomethacin (10 mg/kg, *p.o.*)	Formulation (300 mg/kg, *p.o.*)	Formulation (600 mg/kg, *p.o.*)
0	0	0	0	0
1	0.55±0.03	0.33±0.06* (40.00)	0.38±0.03 (31.82)	0.30±0.05* (45.56)
2	0.65±0.04	0.35±0.05* (44.00)	0.43±0.02* (32.00)	0.38±0.03* (40.00)
3	0.78±0.06	0.30±0.05* (61.29)	0.38±0.03* (51.61)	0.35±0.05* (54.84)
4	0.68±0.06	0.28±0.04* (59.26)	0.38±0.03* (44.44)	0.33±0.02* (51.85)

Values are expressed as mean±SEM, n=6 in each group. Values in parentheses indicate percent inhibition of paw edema.

* *P*<0.01 as compared with control.

**TABLE 2:** EFFECT OF FORMULATION ON COTTON PELLET GRANULOMA IN RATS

Treatment	Dry weight of granuloma† (mg)
Control (Vehicle, *p.o.*)	48.80±0.89 (-)
Indomethacin (5 mg/kg, *p.o.*)	20.88±0.63* (57.08)
Formulation (300 mg/kg, *p.o.*)	35.13±0.51* (27.92)
Formulation (600 mg/kg, *p.o.*)	22.78±0.43* (53.17)

† Difference between weight of dissected dry cotton pellet and weight of cotton pellet before implantation. Values are expressed as mean±SEM, n=6 in each group. Values in parentheses indicate percent inhibition of granuloma formation.

* *P*< 0.01 as compared with control.

The antiinflammatory effect of the formulation on carrageenan-induced rat paw edema is presented in Table 1. The development of carrageenan-induced edema is biphasic, the 1^st^ phase is mediated through the release of histamine and serotonin, with a peak value at 1h; whereas the 2^nd^ phase is related to the release of prostaglandins with a peak value at 3 h^13^,^14^. Edema suppressant effect of 300 mg/kg and 600 mg/kg treated groups were found to be significant on both the phases of inflammation when compared to control. The 300 mg/kg and 600 mg/kg doses showed maximum anti-edematous effect of about 51.61% and 54.84%, respectively at 3 h after carrageenan administration which was found to be significant statistically (*P*<0.01) as compared to control. Indomethacin (standard) showed similar type of reduction at 3 h (*P*<0.01) as compared to control. Since the formulation in our study exhibited significant inhibitory effect on the rat paw edema, it probably exerts an inhibitory effect on some of the mediators of inflammation induced by the carrageenan stimuli. Swingle and Shildeman demonstrated that there are three phases of inflammation after pellet implantation^15^; consisting of a transudative phase, defined as the increase in wet weight of the pellet which occurs during the first 3 h (passive process), an exudative phase which occurs between 3 and 72 h after implanting the pellet (a delayed-prolonged vascular permeability change in inflammation) and a proliferative phase, measured as the increase in dry weight of granuloma which occurs between 3 and 6 d after implantation^15^. In our study, the antiinflammatory activity was indicated by a significant reduction in the granular tissue formation. The animals when treated with formulation at doses of 300 mg/kg and 600 mg/kg orally showed a significant (*P*<0.01) inhibition of granuloma formation of 27.92% and 53.17%, respectively and indomethacin (5 mg/kg, *p.o.*) showed 57.08% inhibition as compared to control. In conclusion the formulation exhibits remarkable antiinflammatory activity in the acute phase as well as in the subacute model of inflammation.
